# Oral Presentations

**DOI:** 10.1080/135771402320883862

**Published:** 2002-09


### ORAL PRESENTATIONS

(In Program order)

**Friday, November 1**

The Role Of Ultrasonography To Evaluate Chest Wall Sarcomas
[Abstract ID: 39]

**Category:** Surgery
**Presentation:** Oral
**Authors:** Antonio Briccoli[1], Michele Rocca[1], Stefano Galletti[2], Elio Ricchi[3]
**Author Institutions:** [1]Department of General Surgery Rizzoli Orthopaedic Institute Bologna, Italy; [2]Department of Ultrasound Imaging Rizzoli Orthopaedic Institute Bologna, Italy; [3]General Surgery University of Modena, Italy
**Presenter:** Antonio Briccoli
antonio.briccoli@ior.it
**Correspondent:** Antonio Briccoli
antonio.briccoli@ior.it
Bologna Italy 40136
Ph: 390516366222
Fax: 39051331710

*Objectives:* Surgery is the elective treatment for chest wall sarcoma. Local recurrence, varies from 7% (Pairolero) to 52% (King), and is normally determined by an inadequate surgical exeresis.
The goal of this paper is to define a preoperative approach to study tumor margins.
*Methods:* 19 patients with a chest wall sarcoma were routinely submitted to an Rx, CT, MRI, total body scintigraphy, and US evaluation. US was always performed by the same person with a Siemens Sonoline Omnia, employing a probe 5–13 Mhz, and performing a color-doppler evaluation of the lesion. The scan was done with the patient lying in the surgical position. Tumor lateral margins were identified and a line was marked out at 4 cm (safe oncological margin).
*Results:* Histologically all the surgical specimen removed following the US margins revealed wide exeresis (Enneking classification). On the contrary CT scan and MRI showed a 27% underestimation and a 18% overestimation respectively of the tumor lateral margins. CT and MRI correctly evaluated in 94% of patients deep margins.
US detected one or more micronodules in 4 out of 5 patients with local recurrence. Histologically they were all sarcoma nodules (mean diameter 3 mm).
18 patients were disease free at 24 months (4–9). One patient died due to cerebral and bone metastasis.
*Conclusions:* US sensitivity (100%), specificity (98%), and accuracy (98%) in detecting chest wall involvement of lung cancer is well known since 10 years (Suzuki). Our results confirm the reliability of US also in the study of chest wall sarcomas superficial margins and micronodules.

High-resolution Intravascular Ultrasonography To Assess Vessel-invasiveness Of Soft Tissue Sarcoma
[Abstract ID: 78]

**Category:** Surgery
**Presentation:** Oral
**Authors:** Peter Hohenberger[1], Michael Huenerbein[1]
**Author Institutions:** [1]Division of Surgery and Surgical Oncology Charite Humboldt University at Berlin, Germany
**Presenter:** Peter Hohenberger
hohenberger@rrk-berlin.de
**Correspondent:** Peter Hohenberger
hohenberger@rrk-berlin.de
Berlin, Germany D-13125
Ph: +49–30–9417–1406
Fax: +49–30–9417–1439

*Objectives:* Detecting the extension of local spread of soft tissue sarcoma (STS) is of utmost importance for adequate resection. However, even MRI fails in correctly describing tumor invasion to neurovascular bundles. We explored, whether high-resolution (intravascular ultrasound (IVUS) improves correct assessment of vascular infiltration.
*Methods:* To qualify for enrollment, patients had to have a histologically confirmed STS with the tumor located close to and suspected to invade a neurovascular bundle.
Twenty-eight patients (12 f, 16 m, age 30–72 yrs) were examined. Tumors were located in the retroperitoneum (n=4), groin (n=7), thigh (n=9), popliteal fossa (n=9), lower leg (n=2), and axilla (n=1). Typing: liposarcoma (n=8), leiomyos. (n=6), MFH (n=6), synovial (n=5), MPNST (n=3), alveolar (n=1), hemangiopericytoma (n=1).
A 2.9F IVUS-catheter (12.5 MHz, Ultracross, Boston Scientific Corp.) was inserted via a side branch of the vessels exposed distant to the tumor during resection. IVUS findings were compared with intraoperative assessment and the resection specimen.
*Results:* In 16 patients, the vessels were resected, while 2 patients underwent subadventitial dissection, and in 10 patients the vessels were left untouched. IVUS detected 13 cases of vessel-invasive sarcoma (true positives). There were two false positives and one false negative whereas in another 12 cases no invasion could be found (true negative). Sensitivity: 92.8%, Specificity: 85.7%, positive predictive value (PPV): 86.6%, negative predictive value (NPV): 92.3%.
*Conclusions:* IVUS provides an excellent tool to assess vessel invasion of STS. It can be handled intraoperatively and by this way allows the surgeon to examine the region of interest exactly. High-resolution ultrasound might overcome some of the problems of local staging still being not solved by MRI.

Predictive Value Of Gadolinium Enhancement In Differentiating Atypical Lipomas (well-differentiated Liposarcomas) From Benign Fatty Tumors
[Abstract ID: 27]

**Category:** Diagnostic Radiology
**Presentation:** Oral
**Authors:** Matthew J. Panzarella[1], Timothy A. Damron[1]
**Author Institutions:** [1]SUNY Upstate Medical University at Syracuse, New York, United States **Presenter:** Matthew J. Panzarella
panzarem@mail.upstate.edu
**Correspondent:** Timothy A. Damron
tdamron@twcny.rr.com
Syracuse New York United States 13202
Ph: 315–464–4472
Fax: 315–464–4664

*Objectives:* To determine the predictive value of gadolinium enhancement on MRI in differentiating atypical lipomas (well-differentiated liposarcomas) from benign fatty tumors.
*Methods:* Of 129 patients evaluated in the office of the senior author for fatty tumors over the period 1994–2002, the patient population was narrowed to 34 histologically proven fatty tumors with pre-op gadolinium-enhanced MRI, 14 of whom had undergone biopsy after the MRI. Pre-operative gadolinium enhancement was based upon consensus of the musculoskeletal surgeon and radiologists prior to definitive operation. Sensitivity, specificity, positive and negative predictive values for both gadolinium enhancement and biopsy in predicting final atypical lipoma diagnosis (considered equivalent to well-differentiated liposarcoma) were calculated.
*Results:* As a predictor of atypical lipoma, gadolinium enhancement showed 100% sensitivity , 71% specificity , 59% positive predictive value, and 100% negative predictive value. Needle or incisional biopsy yielded 67% sensitivity, 100% specificity , 100% positive predictive value, and 63% negative predictive value.
*Conclusions:* Gadolinium enhancement of a homogenous fatty soft tissue tumor, when determined by consensus of an experienced musculoskeletal team, is a sensitive screening tool to determine possible diagnosis of atypical lipoma. Biopsy, on the other hand, is specific but insensitive. An algorithm is proposed for evaluation of these tumors in which all deep or large tumors undergo screening with gadolinium-enhanced MRI. Only those tumors that enhance would be considered for directed biopsy.

Fdg- Positron Emission Tomography (pet) In Staging And Managing Patients With Soft Tissue Sarcomas
[Abstract ID: 61]

**Category:** Diagnostic Radiology
**Presentation:** Oral
**Authors:** Thomas F. DeLaney[1], Francis J. Hornicek[1], Mark C. Gebhardt[1], Mark J. Ott[1], Alan Fischman[1], Peter A. Chapman[1], Andrew R. Rosenberg[1], Daniel I. Rosenthal[1], David C. Harmon[1], Herman D. Suit[1]
**Author Institutions:** [1]Massachusetts General Hospital, MA, United States
**Presenter:** Thomas F. DeLaney
tdelaney@partners.org
**Correspondent:** Thomas F. DeLaney
tdelaney@partners.org
Boston MA United States 02114
Ph: 617–726–7869
Fax: 617–724–9532

*Objectives:* FDG (2–18fluoro-2-deoxy-D-glucose) PET scanning is useful for oncologic staging and treatment assessment. We evaluated its role in patients with soft tissue sarcomas.
*Methods:* 59 patients underwent 97 PET scans, 50 at initial staging, which included primary site MRI and chest CT. PET activity was scored as 0=normal, 1= minimal, 2=moderate, and 3= intense. PET results were compared with chest CT to assess sensitivity /specificity. Most patients underwent neoadjuvant radiation or chemoradiation followed 3–4 weeks later by surgery. Re-staging PET, MRI, and chest CT were performed 1–3 weeks after neoadjuvant therapy and PET activity in the primary site was correlated to residual tumor in the surgical specimen.
*Results:* Increased activity was seen at the primary site in 47 patients (94 %); median and mean activity scores were respectively 3 and 2.75. Two patients without PET activity had undergone excisional biopsy; a third had grade 1 liposarcoma. Sensitivity and specificity for PET for detecting lung metastases were 0.09 and 0.97 respectively, compared to 0.75 and 0.83 for chest CT. Positive and negative predictive values for PET were 0.50 and 0.77 compared to 0.56 and 0.92 for chest CT. On 39 re-staging PETs after neoadjuvant treatment, there was less activity, median score 2 and mean 2.18. No clear relationship emerged between residual activity and percent necrosis in the surgical specimen, although this has not yet been corrected for tumor grade.
*Conclusions:* Soft tissue sarcomas are FDG avid at the primary site; activity decreases with neoadjuvant therapy. PET scanning is not useful for detection of lung metastases.

Fluorodeoxyglucose Uptake In Adult Soft Tissue Sarcoma Predicts Risk Of Recurrence After Chemotherapy
[Abstract ID: 31]

**Category:** Medical Oncology
**Presentation:** Oral
**Authors:** Scott M Schuetze[1], Brian P Rubin[1], Cheryl Vernon[1], James D Bruckner[1], Ernest U Conrad III[1], Janet F Eary[1]
**Author Institutions:** [1]University of Washington, Seattle, Washington, United States
**Presenter:** Scott M Schuetze
sschuetz@fhcrc.org
**Correspondent:** Scott M Schuetze
sschuetz@fhcrc.org
Seattle Washington United States 98109
Ph: 206 288–2052
Fax: 206 288–2042

*Objectives:* Patients with localized, high-grade soft tissue sarcomas (STS) are at significant risk of developing metastasis. Chemotherapy may reduce the risk of relapse; however, not all patients benefit from treatment. High-grade STS metabolize fluorodeoxyglucose (FDG) to a greater extent than normal tissue which can be quantified by positron emission tomography (PET). We sought to determine whether the change in metabolic activity of STS in response to chemotherapy could serve as a surrogate measure for chemosensitivity and correlate with risk of disease recurrence.
*Methods:* FDG-PET was performed prior to doxorubicin-based chemotherapy and prior to surgery. Patients were followed for a minimum of 2 years for recurrence of disease and death. Patient outcomes were analyzed by Kaplan-Meier analyses stratified by change in FDG uptake, tumor grade, tumor size and pathologic response.
*Results:* Thirty-eight patients with localized, high-grade STS were retrospectively studied. Seventeen patients remain free of tumor with a median follow-up of 3 years. Patients with a greater than 40% decrement in the maximum FDG uptake in STS in response to chemotherapy had a significantly lower risk of relapse and improved survival. Risk of relapse did not correlate with tumor grade, size or pathologic response to therapy.
*Conclusions:* A change in the metabolism of FDG in response to chemotherapy may serve as a surrogate measure of chemotherapy sensitivity in high-grade soft tissue sarcomas. Patients with a large decrement in FDG uptake in response to doxorubicin-based therapy have a lower risk of disease recurrence and improved survival.

Should Soft Tissue Sarcomas Be Treated At A Specialist Centre?
[Abstract ID: 48]

**Category:** Surgery
**Presentation:** Oral
**Authors:** Robert Grimer[1], Aneel Bhangu[1], John Beard[1]
**Author Institutions:** [1]Royal Orthopaedic Hospital, United Kingdom
**Presenter:** Robert Grimer
rob.grimer@btopenwold.com
**Correspondent:** Robert Grimer
rob.grimer@btopenworld.com
Birmingham United Kingdom B31 2AP
Ph: (+44) 121 685 4150
Fax: (+44) 121 685 4146

*Objectives:* We have investigated whether there is evidence that patients with soft tissue sarcomas do better if treated in a specialist centre compared with district general hospitals.
*Methods:* We analysed the outcomes for all patients with soft tissue sarcomas in one health region of the UK over a 3 year period, with minimum follow up of 5 years. We have investigated appropriateness of treatment, adequacy of surgery, and outcomes in terms of local control and overall survival. Results are stratified for known risk factors for local control and survival (grade, depth and size).
*Results:* 260 patients were diagnosed as having STS over the 3 year period of whom 37% had the majority of treatment at the specialist centre under the care of 2 surgeons, whilst the other 63% were treated at a total of 38 different hospitals. Local recurrence rates were 20% at the specialist centre and 37% at the general hospitals. Overall survival was 58% at five years and was related to grade, depth and size of tumour. Patients treated at the specialist centre had larger tumours (10.3 vs 7.3cm) with a higher proportion of deep and high grade tumours. Overall survival at the two centres was identical but when stratified for known risk factors the survival rate was 1.6 times better at the specialist centre, this difference being especially obvious for Stage III tumours (p = 0.009).
*Conclusions:* Soft tissue sarcomas are rare. Centralization of treatment, especially for high grade tumours improves survival, local control and patients care.

Positive Margins After Attempted Wide Excision Of A Soft Tissue Sarcoma: Is Further Excision Necessary?
[Abstract ID: 55]

**Category:** Surgery
**Presentation:** Oral
**Authors:** Edward Y. Cheng[1], Kathryn E. Dusenbery[1], Carlos M. Manivel[1], Denis R. Clohisy[1], Keith Skubitz[1], Roby C. Thompson[1]
**Author Institutions:** [1]University of Minnesota Cancer Center, Minnesota, United States
**Presenter:** Edward Y. Cheng
cheng002@umn.edu
**Correspondent:** Edward Y. Cheng
cheng002@umn.edu
Minneapolis MN United States 55455
Ph: 612–625–4653
Fax: 612–626–6032

*Objectives:* When the surgical margin is positive after a planned wide excision, the aims of our study were to discern: 1)Is additional surgical excision or amputation warranted and, 2)If no additional surgery is done, is there a difference in overall survival (OS), continuous disease free survival (CDFS) or local recurrence (LRFS)?
*Methods:* We performed a review of all patients meeting the inclusion criteria: non-metastatic soft tissue sarcomas (excluding rhabdo), extremity, trunk, retroperitoneum,treatment period 1975 – 1997 @ Univ. Minnesota, age > 13 years. The treatment protocol was: attempted wide local excision, adjuvant radiation when closest margin is marginal, if positive margin identified on pathological analysis, further excision considered if technically possible depending upon size of surgical bed, anatomic location, patient's desires. Independent pathologist evaluation was performed with surgical margins defined as: Negative (> 2 mm closest margin), Negative (< 2 mm closest margin), Positive-microscopic, Positive-macroscopic (gross tumor remaining), Inadvertent intraop contamination. Patients w/ a positive margin were then compared to those w/ a negative margin using standard survivorship analysis and Cox stepwise regression statistical methods.
*Results:* The study cohort consisted of 413 patients (180 female, 233 male), mean age 51 yrs, with diagnoses liposarcoma (104), MFH (142), synovial sarcoma (44), other sarcoma (123). Tumor grades were low (50), intermediate(53), & high (254). External beam radiation, mean dose 5688 cGy, was given preoperatively w/ postop boost to 112 (27%)patients & postoperatively only to 170 (41%) patients. No radiation was given to 131 (32%) patients. There was no difference in 5 yr OS between the positive vs. negative margin group, 76% vs. 61% (p=0.4), respectively. There was no difference in 5 yr CDFS between the positive vs. negative margin group, 61% vs. 68% (p=0.21), respectively. There was a statistical difference in 5 yr LRFS with the negative margin group being higher, 88% vs. 75% (p=0.02). The influence of grade and margin revealed that for LRFS, positive margin was a risk factor (p=0.02) but grade was not. For both CDFS and OS, grade was a risk factor (p=0.01 and 0.04, respectively) but margin status was not. Among only low grade tumors there was no diff in OS, CDFS or LRFS comparing the positive vs. negative margin groups. Among only high grade tumors, there was a diff in LRFS (p=0.002) and CDFS (p=0.049) but not OS.
*Conclusions:* We conclude a positive margin is associated with a higher risk of local relapse but not necessarily survival. When a positive surgical margin is sustained after a planned attempt at wide excision, additional surgery is not warranted for low grade tumors. For high grade tumors, CDFS but not OS is shorter when a positive margin occurs and therefore, it is uncertain if this is significant enough to warrant additional ablative surgery or amputation.

Microarray Analysis Of Malignant Fibrous Histiocytoma
[Abstract ID: 73]

**Category:** Biology
**Presentation:** Oral
**Authors:** Jay S Wunder[1], Nalan Gokgoz[2], Sasha Eskandarian[2], Wenqing He[2], Shelley B Bull[2], Robert E Turcotte[5], Vivien H Bramwell[4], Robert S Bell[1], Rita Kandel[3], Irene L Andrulis[2]
**Author Institutions:** [1]University Musculoskeletal Oncology Unit Mount Sinai Hospital Toronto, Ontario, Canada; [2]Samuel Lunenfeld Research Institute Mount Sinai Hospital Toronto, Ontario, Canada; [3]Department of Pathology Mount Sinai Hospital Toronto, Ontario, Canada; [4]London Regional Cancer Centre London, Ontario, Canada; [5]University of Montreal Montreal, Quebec, Canada
**Presenter:** Jay S Wunder
wunder@mshri.on.ca
**Correspondent:** Jay S Wunder
wunder@mshri.on.ca
Toronto Ontario Canada M5G 1X5
Ph: 416–586–8807
Fax: 416–586–8397

*Objectives:* Malignant fibrous histiocytoma (MFH) is the most common type of soft tissue sarcoma but is poorly understood. There are few accurate predictors of outcome to guide treatment decisions. We used microarray analysis of gene expression to identify prognostic markers for MFH.
*Methods:* 40 MFH tumor specimens with clinical data were chosen from a prospective tumor bank from patients who did not receive preoperative chemotherapy or radiotherapy. Frozen specimens were assessed histologically to confirm viable tumor. Tumor and control RNA were indirectly labelled with fluorescent tags and simultaneously hybridized to 19K microarray slides. Arrays were scanned, quantitated and normalized prior to statistical analysis using GenePix and SPlus, and then analysed with BrB ArrayTools.
*Results:* We selected 111 "candidate" genes from a variety of biological pathways with potential importance in MFH, and examined their ability to discriminate between clinicopathologic variables, stage, and oncologic outcome. For each clinical variable, a group of 5–13 genes were identified which distinguished between categorical strata (F-test $p<0.05$). For example, 10 genes differentiated between patients who did or did not develop metastases, including TOK1 (p21-binding protein), SEI1 (cdk4-binding protein), CA1A (collagen-related gene), IRF1 (interferon regulatory factor1) and MMP9. However, rigorous assessment of prediction error using cross-validation techniques suggested that combinations of the above genes did not significantly improve prediction, recognizing the low power and small size of this sample.
*Conclusions:* This study suggests that the candidate gene list approach does not provide the most accurate method for class prediction for MFH. We are presently undertaking tumor comparison using an expanded microarray 19K gene set without predetermined gene selection which will likely be a more powerful approach to identify prognostically important genes in MFH.

Genome-wide Analysis Of Gene Expression In Synovial Sarcoma Using A Cdna Microarray
[Abstract ID: 49]

**Category:** Pathology
**Presentation:** Oral
**Authors:** Junya Toguchida[1], Satoshi Nagayama[2], Yusuke Nakamura[2], Nobuhito Araki[5], Katsuyuki Kusuzaki[4], Tomitaka Nakayama[3], Takashi Nakamura[3]
**Author Institutions:** [1]Institute for Frontier Medical Sciences Kyoto University, Kyoto, Japan; [2]Institute of Medical Science The University of Tokyo, Tokyo, Japan; [3]Department of Orthopaedic Surgery Kyoto University, Kyoto, Japan; [4]Department of Orthopedic Surgery Kyoto Prefectural University of Medicine, Kyoto, Japan; [5]Department of Orthopedic Surgery Osaka Medical Center for Cancer and Cardiovascular Diseases, Osaka, Japan
**Presenter:** Junya Toguchida
togjun@frontier.kyoto-u.ac.jp
**Correspondent:** Junya Toguchida
togjun@frontier.kyoto-u.ac.jp
Kyoto Kyoto Japan 606–8507
Ph: +81–75–751–4134
Fax: +81–75–751–4134

*Objectives:* Among soft tissue sarcomas, synovial sarcoma (SS) is regarded as a miscellaneous entity of uncertain origin. Although the presence of SYT-SSX fusion gene is a hallmark of this type of tumor, its genetic features remain largely unclear.
*Methods:* We examined genome-wide gene expression profiles of 13 SS cases and 34 other spindle cell sarcoma cases, including MFH, leiomyosarcoma (LMS), pleomorphic or dedifferentiated liposarcoma (PLS or DLS), and malignant peripheral nerve sheath tumor (MPNST), by means of cDNA microarray consisting of 23,040 genes.
*Results:* A hierarchical clustering analysis using the data of 1,204 genes revealed that MFH, LMS, PLS, and DLS failed to compose a disease-specific cluster. On the other hand, SS cases showed a distinct cluster along with MPNST. Four biphasic tumors were clustered closely together, whereas tumors with monophasic features failed to make one cluster. 26 genes were identified as genes that were commonly upregulated in SS, of which most were also upregulated in MPNST, and the presumed function of known genes among them were related to migration or differentiation of neural crest cells, a strong indication that SS originates in the neuroectoderm. On the basis of the expression patterns of the 1,405 genes, 13 SS tumors were subdivided into two distinct subclasses. 15 additional SS cases were also successfully subdivided into either of two groups, suggesting the novel subclassification of SS based on the gene expression profiles.
*Conclusions:* These data provide us with the information for the origin of SS, and also several candidate genes for molecular therapeutic targets.

Differential Gene Expression In Leiomyosarcoma.
[Abstract ID: 24]

**Category:** Biology
**Presentation:** Oral
**Authors:** Keith M Skubitz[1], Amy P. Skubitz[1]
**Author Institutions:** [1]University of Minnesota, MN, United States **Presenter:** Keith M Skubitz
skubi001@tc.umn.edu
**Correspondent:** Keith M Skubitz
skubi001@tc.umn.edu
Minneapolis MN United States 55455
Ph: 612–625–5411
Fax: 612–626–1441

*Objectives:* Soft tissue sarcomas are a heterogeneous group of malignancies of mesenchymal origin, the causes of which are unclear. In this study gene expression in leiomyosarcomas, myometrium, and uterine leiomyomas was examined.
*Methods:* RNA was prepared and gene expression was determined at Gene Logic Inc. (Gaithersburg, MD) using Affymetrix GeneChip® U_95 arrays containing approximately 12,000 known genes and 48,000 ESTs. Gene expression analysis was performed with Gene Logic Gene Express® software. Differences in gene expression were quantified as the fold change in gene expression in leiomyosarcomas compared with normal myometrium, and uterine leiomyomas.
*Results:* A number of genes were differentially expressed in these sample sets, and these genes were analyzed for their expression in a variety of other normal and diseased tissues. Some of the genes identified were over-expressed only in leiomyosarcomas among the tissues examined, and some were over-expressed in other tissues as well
*Conclusions:* We conclude that differences in gene expression can be detected between leiomyosarcomas and uterine leiomyomas, and normal myometrium, and that these changes in gene expression may yield clues to the pathophysiology of this malignancy.

Antisense Inhibition Of Hyaluronan Synthase-2 In Human Osteosarcoma Cell Line, Mg-63, Inhibits Hyaluronan Retention And Tumorigenicity Of The Cells
[Abstract ID: 35]

**Category:** Biology
**Presentation:** Oral
**Authors:** Yoshihiro Nishida[1], Warren Knudson[2], Cheryl B Knudson[2], Izuru Tabata[1], Naoki Ishiguro[1]
**Author Institutions:** [1]Department of Orthopaedic Surgery Nagoya University School of Medicine, Japan; [2]Department of Biochemistry Rush Medical College, IL, United States
**Presenter:** Yoshihiro Nishida
ynishida@med.nagoya-u.ac.jp
**Correspondent:** Yoshihiro Nishida
ynishida@med.nagoya-u.ac.jp
Nagoya Japan 466–8550
Ph: +81–52–741–2111
Fax: +81–52–744–2260

*Objectives:* Osteosarcoma is the common primary malignant tumor of bone and is represented by a heterogenous group of lesions with diverse histopathology and clinical behavior. Hyaluronan (HA) has been thought to play significant roles in tumor progression. The aim of this study was to determine the differential pattern of expression of three hyaluronan synthase genes (HAS), that are responsible for the synthesis of HA in osteosarcomas. An additional goal was to analyze the effects of the inhibition of HAS genes by means of antisense oligonucleotides on the behavior of osteosarcoma cells.
*Methods:* Total RNA was isolated from osteosarcoma cell lines, MG-63, which were cultured in monolayer, and subjected to competitive, quantitative RT-PCR to determine the mRNA copy numbers of HAS genes. Antisense oligonucleotides complimentary to the predominant HAS gene was transfected into osteosarcoma cells using a lipofection facilitator. The inhibition of HAS

and HA expression was determined competitive RT-PCR and HA staining, respectively. Cell proliferation and invasion were analyzed by MTT assay and matrigel assay.
*Results:* HAS-2 mRNA expression was 90-hold higher than HAS-3. Antisense oligos treatment for HAS-2 resulted in a 60% inhibition of HAS-2 mRNA levels and a remarkable decrease of HA accumulation around the cells. The suppression of HAS-2 also lead to the suppression of cell proliferation and invasion.
*Conclusions:* HA inhibition via the down-regulation of HAS genes could control the behavior of osteosarcoma cells. Thus, the use of small antisense oligonucleotides to affect the selective inhibition of genes may provide a useful tool for the control of osteosarcomas.

Neoplastic Transformation Of Primary Human Osteoblasts Over-expressing The Met Receptor By Means Of Transduction With Lentiviral Vectors
[Abstract ID: 22]

**Category:** Biology
**Presentation:** Oral
**Authors:** Riccardo Ferracini[1], Nicola Baldini[3], Nadia Coltella[1], Sofia Avnet[3], Salvatore Patane'[1], Martina Olivero[1], Luigi Naldini[2], MariaFlavia Di Renzo[1]
**Author Institutions:** [1]Laboratory of Cancer Genetics of the Institute for Cancer Research and Treatment IRCC Candiolo, Italy; [2]Laboratory of Gene Therapy of the Institute for Cancer Research and Treatment IRCC Candiolo, Italy; [3]Laboratory for Pathophysiology of Orthopaedic Implants Istituti Ortopedici Rizzoli Bologna, Italy **Presenter:** Riccardo Ferracini
rferracini@ircc.unito.it
**Correspondent:** Riccardo Ferracini
rferracini@ircc.unito.it
Candiolo Italy 10060
Ph: +39 011 9933 460
Fax: +39 011 9933 225

*Objectives:* The MET oncogene is activated in cell lines where it is amplified and over-expressed. Point mutations of its kinase domain leading to its activation have been found in families suffering from hereditary papillary renal cell carcinomas. We previously showed that the receptor is aberrantly expressed in a number of human osteosarcomas producing the ligand, suggesting that overexpression of MET receptor(s) might contribute to transformation of osteoprogenitor cells.
*Methods:* To demonstrate the transforming potential of MET in human osteoblast-like cells(HOB-L), we constructed an ex-vivo model using primary cultures of HOB-L stably expressing wild-type or constitutively-active MET receptors by means of transduction with Lentiviral vectors in vitro.
*Results:* All the wt- and mutant-MET-HOB-L clones showed a different morphology from parental HOB-L cells, namely a loss of contact-inhibition, proliferation at low serum concentrations and anchorage-independent growth. They formed colonies in soft agar, while the parental HOB-L cells did not.
Tumorigenic potential of MET-HOB-L cells was assayed in immuno-compromised SCID mice. Two out 12 animals, which received wt-MET transduced osteoblast cells, developed tumor after a long latency. Nine out of 16 mice receiving mutated MET-expressing osteoblast cells developed tumors in a shorter period. These tumors showed features of highly aggressive and undifferentiated osteosarcomas and carried integrated copies of the human MET.
*Conclusions:* Over-expression of the MET oncogene activated by a point mutation makes the same cells not only transformed but also highly tumorigenic in immuno-compromised mice. These results show that over-expression of an activated tyrosine kinase suffices to convert a primary human cell into a tumorigenic cell.

**Young Investigator Award Winner**

Targeting Met Oncogene In Human Osteosarcoma By K-252a Tyrosine-kinase Inhibitor: A New Therapeutic Approach
[Abstract ID: 71]

**Category:** Biology
**Presentation:** Young Investigator
**Authors:** Sofia Avnet[1], Francesca Perut[1], Riccardo Ferracini[2], Maria Flavia Di Renzo[2], Nicola Baldini[1]
**Author Institutions:** [1]Laboratory of Pathophysiology Istituto Ortopedico Rizzoli Bologna, Italy; [2]Laboratory of Cancer Genetics Institute for Cancer Research and Treatment Candiolo, Italy
**Presenter:** Sofia Avnet
sofia.avnet@ior.it
**Correspondent:** Nicola Baldini
nicola.baldini@ior.it
Bologna Italy 40136
Ph: +39.(0)50.6366678
Fax: +39.(0)50.6366748

*Objectives:* Activation of Met, the tyrosine kinase receptor for HGF/SF, induces downstream signalling pathways that trigger a number of cell functions, including proliferation, motility, invasiveness, and metastatic ability in a variety of neoplasms. Met is overexpressed in osteosarcoma, in association with an aggressive behavior of this tumor, both in vitro and in a clinical setting. We evaluated the ability of a natural alkaloid (K252a) which acts as a kinase inhibitor, by competing with the binding of ATP to the catalitic domain of Met, to interfere with the growth of human osteosarcoma cells, as a preliminary step toward the development of innovative therapeutic strategies for this tumor.
*Methods:* We evaluated the level of Met expression by Western blotting in human osteosarcoma cell lines (U-2 OS, MG-63, Saos-2), that are representative of different levels of differentiation and aggressiveness of osteosarcoma. IC50 was analyzed at different concentrations of K252a (50–2000 ng/ml).
*Results:* In osteosarcoma cells, the level of Met expression was associated with the ability of K252a to inhibit cell growth. In fact, the IC50 of K252a was 420 ng/ml in Saos-2, the more differentiated cell line with the lowest expression of Met, whereas it was 55.8 ng/ml in U-2 OS, the cell line with the lowest differentiated phenotype and the highest Met levels. Intermediate values (IC50, 190.7 ng/ml) were found in MG63.
*Conclusions:* K252a is a potent inhibitor of proliferation of human osteosarcoma, and its effects are positively associated with the level of expression of Met, but inversely related to the differentiative status of the tumor.

Differential Effects Of The Radioprotectant Amifostine On Ewing's Sarcoma And Human Monocytes In Culture
[Abstract ID: 46]

**Category:** Biology
**Presentation:** Oral
**Authors:** Bryan Margulies[1], Timothy A. Damron[1], Matthew J. Allen[1]
**Author Institutions:** [1]SUNY Upstate Medical University at Syracuse, New York, United States **Presenter:** Matthew J. Allen
allenm@mail.upstate.edu
**Correspondent:** Timothy A. Damron
tdamron@twcny.rr.com
Syracuse New York United States 13202
Ph: 315-464-4472
Fax: 315-464-4664

*Objectives:* Radioprotectants must be selectively radioprotectant, protecting normal cells at the expense of tumor cells. Such selectivity has not been established for sarcomas. The hypothesis investigated in this pilot was that amifostine alone would not promote proliferation of Ewing's sarcoma cells or human monocytes.
*Methods:* Amifostine at concentrations of 0, 0.5, 1, 2, 5, and 10 mM was administered to confluent TC-71 Ewing's sarcoma cells. At 6, 12, 24, and 72 hours following amifostine administration, MTT assays of cellular proliferation were performed. Clonogenicity assays of reproductive survival were also accomplished utilizing 0, 1, 5, and 10 mM concentrations of amifostine. Human monocytes were cultured from proximal femoral bone marrow aspirated during routine total hip procedures following IRB approved patient consent. These monocytes were similarly assayed following amifostine administration ANOVA was utilized with alpha 0.05.
*Results:* Amifostine was acutely toxic to TC-71 Ewing's sarcoma cells at 5–10 mM, moderately toxic at 2 mM, and mild to moderately toxic at 0.5 and 1 mM as manifested by the MTT assays. Clonogenicity assays on the amifostine treated TC-71 cells confirmed the observations made with the MTT assay. By contrast, human monocytes exhibited a consistent increase in proliferation following amifostine administration.
*Conclusions:* Amifostine has a cytotoxic effect on this Ewing's sarcoma cell line at clinically relevant concentrations (1–2 mM). By contrast, a proliferative effect of amifostine was observed in our human monocyte culture. These effects are promising and warrant further study of the putative differential radioprotective properties of amifostine in sarcomas.

A Randomized Trial Of Amifostine With High-dose Alkylator Therapy In Ewing Sarcoma: A Children's Oncology Group Trial (p9457)
[Abstract ID: 37]

**Category:** Pediatric Oncology
**Presentation:** Oral
**Authors:** Mark L Bernstein[1], Meenakshi Devidas[1], Paul Meyers[1], Dominique Lafreniere[1], Allen Goorin[1], Holcombe Grier[1]
**Author Institutions:** [1]Childrens Oncology Group, United States
**Presenter:** Mark L Bernstein
bernstm@magellan.umontreal.ca
**Correspondent:** Mark L Bernstein
bernstm@magellan.umontreal.ca
Montreal Quebec Canada H3T 1C5
Ph: 514–345–4969
Fax: 514–345–4792

*Objectives:* To determine if amifostine given twice with each dose of ifosfamide or cyclophosphamide could provide protection from the myelotoxic effects of high dosage alkylator therapy, and prevent delay in the administration of chemotherapy.
*Methods:* Patients (pts) less than 30 years of age with Ewing sarcoma metastatic at diagnosis and normal organ function were enrolled on a study of maximally intensified alkylator therapy without stem cell support. Cycles included alternating courses of ifosfamide (3.6 g/m2/day,for 5 days, week 6, then 2.8 g/m2/day,for 5 days, weeks 12 and 18), with etoposide (100 mg/m2/day,for 5 days) and vincristine (2 mg/m2), doxorubicin (75 mg/m2 over 48 hours) and cyclophosphamide (2.1 g/m2/day, for 2 days, weeks 9 and 15). Seventy pts were randomized to receive (36 pts), or not to receive (34 pts) amifostine. Topotecan(0.75 mg/m2/day,for 5 days) and cyclophosphamide (250 mg/m2/day,for 5 days) were administered to 23 patients on the amifostine arm (weeks 0, 3)and 16 on the no amifostine arm. The amifostine dosage administered was 825 mg/m2 15 minutes prior to, and 3 hours after the beginning of each dose of ifosfamide or cyclophosphamide, with premedications and precautions as previously described. Days ANC < 500/ul, days platelets < 50,000/ul and days between cycles were analyzed at weeks 6, 12 and 18 of therapy.
*Results:* There was no significant difference seen in any of the parameters studied: the mean number of days with platelet count < 50,000/ul:Wilcoxon rank sum test (WRS test, p-value: 0.29), the mean number of days with absolute neutrophil count < 500/ul: (WRS, p = 0.15), or the number of days until the next chemotherapy cycle: (p= 0.42). Toxicities attributable to amifostine included increased emesis, reversible hypotension, and asymptomatic hypocalcemia responding to calcium supplementation.
*Conclusions:* In the dose and schedule used, amifostine did not provide myeloprotection from the toxicity of high dose alkylator therapy, and did not shorten the interval until the administration of the next chemotherapy cycle.

Randomised Phase 3 Trial Of Two Investigational Schedules Of Ifosfamide Versus Standard Dose Doxorubicin In Patients With Advanced Or Metastatic Soft Tissue Sarcoma (asts).
[Abstract ID: 20]

**Category:** Medical Oncology
**Presentation:** Oral
**Authors:** Paul Lorigan[1], Jaap Verweij[1], Z Papai[1], S Rodenhuis[1], A Le Cesne[1], M Leahy[1], John Radford[1], Pancras Hogendoorn[1], Anne Kirkpatrick[1], Ole S Nielson[1]
**Author Institutions:** [1]EORTC Soft Tissue and Bone Sarcoma Group, Brussels, Belgium
**Presenter:** Paul Lorigan
paul.lorigan@christie-tr.nwest.nhs.uk
**Correspondent:** Paul Lorigan
paul.lorigan@christie-tr.nwest.nhs.uk
Manchester United Kingdom M20 4BX
Ph: 0161 446 3000
Fax: 0161 446 3299

*Objectives:* Doxorubicin and ifosfamide show significant activity in soft tissue sarcoma but have never been formally compared. We report a randomised Phase III comparison of single agent doxorubicin 75mg/m2 versus ifosfamide 3g/m2 over 4 hours daily for 3 days (Ifos 3*3) versus ifosfamide 9g/m2 over 72 hours by continuous infusion (Ifos 9) in patients with ASTS.
*Methods:* Patients were stratified by histological subtype, grade, metastatic site, performance status and institution. Response was evaluated after each 2 cycles and all responses independently reviewed. An interim analysis was carried out by an independent data monitoring committee after four years.
*Results:* Between 1997 and Sept 2001, 322/760 patients were recruited. Groups were evenly divided by stratification factors. Histological types were leiomyosarcoma 31.4%, liposarcoma 12.1%, synovial sarcoma 9.3%, others 46.2%. Toxicity was worse in both ifosfamide arms; grade 3 febrile neutropenia occurred in 8.3% doxorubicin patients, 20.7% Ifos 3*3 and 17% Ifos 9 patients. Response rates were: Doxorubicin 11% (CR 0.9%, PR 10.1%), Ifos 3*3 6.5% (CR 0.9%, PR 5.6%), Ifos 9 9.4% (CR 1.9%, PR 7.5%). With a median follow up of 14 months, there was no significant difference in progression free or overall survival in the three arms. The study hypothesis of a difference of 10% in PFS at 1 year was rejected and the trial stopped early on the advice of an independent data monitoring committee.
*Conclusions:* For the majority of patients with ASTS for whom single agent chemotherapy is appropriate, doxorubicin 75mg/m2 remains the treatment of choice.

Expression of the Ihh/pthrp and Bmp Receptors Has Diagnostic Relevance in Cartilagineous Lesions
[Abstract ID: 17]

**Category:** Pathology
**Presentation:** Oral

**Authors:** Luca Sangiorgi[1], Joerg Warzecha[1], Enrico Lucarelli[1], Licciana Zanella[1], Marco Alberghini[1], Veronica Maini[1], Elena Pedrini[1], Patrizia Bacchini[1], Franco Bertoni[1], Piero Picci[1]
**Author Institutions:** [1]Rizzoli Orthopedic Institute, Italy
**Presenter:** Luca Sangiorgi
luca.sangiorgi@ior.it
**Correspondent:** Luca Sangiorgi
luca.sangiorgi@ior.it
Bologna Italy 40136
Ph: +39–051–6366519
Fax: +39–051–6366761

*Objectives:* Indian Hedgehog (and its downstream target) and Bone Morphogenic Proteins have shown to play a relevant and independent role in chondrocyte proliferations and regulations during embryonic development. Aim of this study is to evaluate if the expression of these molecules can be used for diagnostic classification of cartilaginous lesions.
*Methods:* Cartilagineous lesions were analysed by immunohistochemistry with the antibodies for Indian Hedgehog (Ihh), Parathyroid Hormone related peptide (PTHrp), Bone Morphogenic Protein Receptors type 1 A (BMPR-IA), type 1B (BMPR-IB) and type 2 (BMPR-II) receptors. Staining was evaluated by 4 investigators independently.
*Results:* We observed a distinct pattern of expression of these proteins in specific subset of cartilagineous lesions. Ihh and BMPR-II expression were found in 4,9% and 5,1% of osteochondromas. In contrast, the same antigens were expressed in 54,1% and 48,3% of peripheral chondrosarcomas. In central chondroid lesions, two different proteins, PTHrp and BMPR-IB expression showed different pattern in enchondromas (11,1% and 25%) and grade I chondrosarcomas (67,7% and 79,5%).
*Conclusions:* Immunohistochemical detection of these proteins could identify different subset of cartilagineous lesions and could be useful for diagnostic purposes.

Dedifferentiated Chondrosarcoma: Updated Outcomes With Current Treatment Approaches As Compared To Those Prior To 1984
[Abstract ID: 19]

**Category:** Surgery
**Presentation:** Oral
**Authors:** Ian Douglas Dickey[1], Sean Patrick Scully[1], Peter S. Rose[1], Laurel Littrell[1], Douglas J. Pritchard[1], Michael George Rock[1], Thomas C. Shives[1], Franklin H. Sim[1]
**Author Institutions:** [1]Mayo Clinic, Minnesota, United States
**Presenter:** Ian Douglas Dickey
dickey.ian@mayo.edu
**Correspondent:** Ian Douglas Dickey
dickey.ian@mayo.edu
Rochester Minnesota United States 55905
Ph: 507–284–0231
Fax: 507–284–5075

*Objectives:* Dedifferentiated chondrosarcoma presents a very difficult clinical problem. Long term survival is known to be poor, but a large clinical series has not been analyzed in the era of modern diagnostic and treatment modalities.
*Methods:* A retrospective chart review of all cases of patients presenting with dedifferentiated chondrosarcoma at our institution from 1984–2000 was performed. This was done as an extension to a study published in 1986 prior to the era of modern chemotherapy.
*Results:* There were 42 cases in 25 men and 17 women of average age 56 (range 24–83 years). MSTS grades at presentation were 5 IIA, 27 IIB, and 10 III. Three patients underwent biopsy only, 19 had limb sacrificing, and 20 had limb sparing procedures; surgical margins were intralesional in 3, marginal in 2, and wide in 20, and radical in 14. Twenty-seven patients received adjuvant therapy (22 chemotherapy only, 2 radiotherapy only, 3 combined therapy). Median survival was 8 months; 5-year survival was 7.1%. There was no statistical difference in survival between patients who did and did not receive chemotherapy, had wide versus radical resection, or had limb sparing versus sacrificing procedures. There were no statistically significant difference between patients treated prior to 1986 and those subsequently.
*Conclusions:* Despite advances in diagnostic modalities, surgical treatments, and adjuvant therapies, dedifferentiated chondrosarcoma continues to carry a poor prognosis. The use of current adjuvant chemotherapy and its inherent risks and benefits remains questionable in this population.

Desmoid Tumors. A Clinical Review Of 30 Patients With More Than 20 Years Follow-up
[Abstract ID: 50]

**Category:** Surgery
**Presentation:** Young Investigator
**Authors:** Mikael Dalén[1], Peter Bergh[1], Björn Gunterberg[1]
**Author Institutions:** [1]Department of Orthopaedic Surgery, Gothenburg, Sweden
**Presenter:** Mikael Dalén
mikaeldalen@hotmail.com
**Correspondent:** Mikael Dalén
mikaeldalen@hotmail.com
Gothenburg Sweden SE-413 45
Ph: 0046 31 3424433
Fax: 0046 31 825599

*Objectives:* Thirty patients with desmoid tumors treated at our department with more than 20 years follow-up (mean 28 years) were reviewed.
*Methods:* Twenty patients were women, 10 were men with a mean age of 39 years. The tumors were located in the abdominal wall (7), upper extremity (6), lower extremity (6), back (5), thoracic wall (4), head-and-neck region (1). One patient presented with multifocal tumors. Largest mean tumor diameter was 7 cm. Twenty-nine patients had surgery, 3 of which had additional radiotherapy. One patient received adjuvant antioestrogen therapy (Tamoxifen®). The surgical margins were intralesional in 6 tumors, marginal in 13 tumors and wide/compartmental in 10 tumors. One patient had radiotherapy alone.
*Results:* Two patients died of non-tumor related causes. Sixteen patients were continuously disease free (CDF). At follow-up, all patients but 1 were free of symptoms. Thirteen patients had no evidence of disease (NED), two of which were primarily operated with debulking surgery. Six patients had mild to moderate post-treatment symptoms. One patient was alive with disease (AWD). She was operated on three times and had additional radiotherapy as well as antioestrogen therapy. At follow-up the patient had disabling symptoms. The overall recurrence rate was 44%. A less tendency for recurrence was evident in patients operated with a wide or compartmental excision. Three tumors disappeared spontaneously.
*Conclusions:* Patients operated with a wide or compartmental margin had the least risk to develop recurrent tumor. Observation alone might be considered in selected cases.

Unexpected Severe Toxicity Of Low Dose Methotrexate And Vinblastine In Patients With Desmoid Tumours
[Abstract ID: 30]

**Category:** Medical Oncology
**Presentation:** Oral

**Authors:** Reginald van der Hul[1], Bert van Geel[1], Caroline Seynaeve[1], Jaap Verweij[1]
**Author Institutions:** [1]Erasmus Medical Center Rotterdam, Netherlands
**Presenter:** Bert van Geel
geel@chih.azr.nl
**Correspondent:** Reginald van der Hul
hul@chih.azr.nl
Rotterdam Netherlands 3075 EA
Ph: +31104391911
Fax: +31104391048

*Objectives:* Evaluation of tolerability of low dose chemotherapy for desmoid tumours.
*Methods:* 10 patients with desmoid tumours (six male; four female; median age 43 years [17–75], median WHO performance score 0 [0–1]), for whom surgery was considered to be severely mutilating, were treated with chemotherapy consisting of weekly intravenous methotrexate 50 mg and vinblastine 10 mg, scheduled to be given for one year. This regimen has previously been reported as active for desmoids and to be devoid of serious toxicity. Toxicity was assessed using NCI-CTC criteria. If full doses could not be given because of side effects the dose was either reduced or the dose interval was extended, based upon the observed type of toxicity. The reasons for preliminary termination of the treatment regimen were registered.
*Results:* No patient could complete the treatment. In all cases dose reduction, delay of dose interval or preliminary termination of the treatment was necessary. All patients had nausea varying from grade I-III, despite antiemetics. Four patients developed polyneuropathy (grade I-III), four had continuous fatigue (grade I-II), two developed leucopenia (grade II-III), two patients developed grade II-III impairment of liver function. One patient developed reversible methotrexate induced pulmonary fibrosis. One patient experienced a complete remission lasting 26 months, eight had stable disease, and one progressed on treatment. Six patients underwent surgery following chemotherapy.
*Conclusions:* Our experience indicates severe long term toxicity is related to this chemotherapy. This is in sharp contrast with a previous report. We believe the regimen can not be recommended for routine use outside study protocols.

Higher Postoperative Radiation Dose Improves The Local Control Rate For Patients With Positive Surgical Margins And Locally Recurrent Soft Tissue Sarcoma
[Abstract ID: 33]

**Category:** Radiation Oncology
**Presentation:** Oral

**Authors:** Matthew T Ballo[1], Gunar K Zagars[1], Shreyaskumar R Patel[1], Peter WT Pisters[1]
**Author Institutions:** [1]UT MD Anderson Cancer Center, United States
**Presenter:** Matthew T Ballo
mballo@mdanderson.org
**Correspondent:** Matthew T Ballo
mballo@mdanderson.org
Houston Texas United States 77030
Ph: 713–792–3400
Fax: 713–794–5573

*Objectives:* To evaluate the relationship between postoperative external beam radiation dose and local control for patients with risk factors for local recurrence after surgery and radiation.
*Methods:* From 1961 to 1999, 804 patients with non-metastatic soft tissue sarcoma were treated with gross total surgical resection and post-operative radiation, with or without adjuvant chemotherapy. Histological sub-type was as follows: MFH, 265 patients; liposarcoma, 97; synovial sarcoma, 91; unclassified, 90; neurogenic sarcoma, 58; Rhabdomyosarcoma, 39; other, 164. Surgical resection margins were positive or uncertain in 377 patients. One hundred and forty eight patients (18%) presented with locally recurrent disease after previous surgical resection. Histological grade was low/intermediate in 234 patients and high in the remaining 570 patients. Median tumor size was 5cm (range 0.5–36cm). Radiation was delivered to a median dose of 61Gy (range 36–75). Only 11 patients received doses <50Gy. Adjuvant systemic therapy was given to 215 patients.
*Results:* At a median follow-up of 12 years, 322 patients (40%) developed disease relapse at any site. The 10-year actuarial overall and disease-free survival rates were 61% and 58%, respectively. The 10-year actuarial local, nodal and distant control rates were 79%, 96%, and 70%, respectively. Multivariate analysis confirmed an independent association between positive surgical resection margins ($p<0.001$), age >47 years ($p=0.002$), high histological grade ($p=0.006$), size >5cm ($p=0.01$), locally recurrent disease ($p<0.001$), postoperative radiation dose <64Gy ($p<0.001$) and an inferior 10-year local control rate. In three of these high-risk subgroups higher radiation dose postoperatively improved the 10-year local control rate. For patients with positive surgical resection margins radiation dose ›64Gy resulted in a 10-year local control rate of 75% compared to 55% for lesser doses ($p<0.001$). For patients presenting with locally recurrent disease after previous surgical treatment radiation doses ›64Gy resulted in a 10-year local control rate of 76% compared to 59% for lesser doses ($p=0.05$). For patients with high grade disease the same doses resulted in 10-year local control rates of 79% as compared to 74%, however this difference was of only borderline significance ($p=0.06$).
*Conclusions:* This radiation dose-response analysis suggests that the adverse prognostic significance of positive surgical resection margins and locally recurrent disease after surgical resection may be partially overcome with postoperative radiation dose in excess of 64Gy.

Advantages Of Proton Beams In Treatment Pelvic Sarcomas
[Abstract ID: 76]

**Category:** Radiation Oncology
**Presentation:** Oral

**Authors:** Herman Suit[1], Thomas Bortfeld[1], Andrzej Niemierko[1], Alexei Trofimov[1], Thomas DeLaney[1]
**Author Institutions:** [1]Massachusetts General Hospital, Massachusetts, United States
**Presenter:** Herman Suit
hsuit@partners.org
**Correspondent:** Herman Suit
hsuit@partners.org
Boston Massachusetts United States 02114
Ph: 617 724 1155
Fax: 617 726 4805

*Objectives:* To assess the clinical gains predicted by use of proton beams rather than the highest technology photon beams in the radiation treatment of sarcomas of the pelvis.
*Methods:* Complete treatment plans based on intensity modulated proton and photon methods for sarcomas at the sacrum, pubic bone/symphysis and inguinal soft tissues. The treatment plans are to account for heterogeneities in tissue densities, motion of the target and critical normal tissues during and between treatment sessions. The comparison of the treatment plans will be based upon calculated complication probabilities for the critical normal tissues for the different plans. Estimates of the maximum dose for each plan for a Grade IV complication probability of 1, 3 and 5% will be presented. Estimates will be presented of the tumor control probability for each plan for the three levels of complication probability.

*Results:* The major predicted clinical gains for each of the three sites are: Site 1 sacrum, virtually no dose to the intestinal track; Site 2 pubic bone/symphysis – virtually no dose to the urinary bladder and Site 3 inguinal regional, virtually no dose to femoral head/neck. The result is marked reduction to treatment related morbidity at those normal tissues. The markedly lower doses to the intestinal tract and bladder will offer the addition gain of higher chemotherapy doses. The final calculated probabilities will be presented in November.
*Conclusions:* For radiation treatment of sarcomas of these three anatomic sites, proton beam technology is predicted to yield a higher proportion of patients who are rendered tumor and complication free.

Phase 2 Trial With Imatinib (glivec, Sti571) In Patients With Gasto-intestinal Stromal Tumors (gist): Activity Results After 1 Year. A Study Of The EORTC Soft Tissue And Bone Sarcoma Group (STBSG).
[Abstract ID: 29]

**Category:** Medical Oncology
**Presentation:** Oral
**Authors:** Jaap Verweij[1], Allan van Oosterom[2], Jean-Yves Blay[3], Ian Judson[4], Sjoerd Rodenhuis[5], Axel Le Cesne[7], Pancras Hogendoorn[6], Martine Van Glabbeke[10], Sasa Dimitrijevic[8], Ole S. Nielsen[9]
**Author Institutions:** [1]Rotterdam Cancer Institute and University Hospital, Netherlands; [2]University Hospital Gasthuisberg Leuven, Belgium; [3]Centre Leon Berard Lyon, France; [4]Royal Marsden Hospital London, United Kingdom; [5]Netherlands Cancer Institute Amsterdam, Netherlands; [6]Leiden University Medical Center, Netherlands; [7]Institut Gustave Roussy Villejuif, France; [8]Novartis Oncology Basle, Switzerland; [9]Aarhus Kommune Hospital, Denmark; [10]EORTC Data Center Brussels, Belgium
**Presenter:** Jaap Verweij
verweij@onch.azl.nl
**Correspondent:** Jaap Verweij
verweij@onch.azl.nl
Rotterdam Netherlands 3075 EA
Ph: +31 10 4391338
Fax: +31 10 4391003

*Objectives:* From January to March 2002, the STBSG has included 27 patients with GIST in a phase II trial of imatinib.
*Methods:* Patients were treated at a dose of 400 mg b.i.d. (the maximum tolerated dose according to the results of the previous STBSG phase I trial in soft tissue sarcoma).
*Results:* Median age was 56 years (range: 30–75), 19 patients were male (70%), performance status (WHO scale) was 0 in 12 cases (44%) and 1 in 15 cases. The reported disease origin was gastro-intestinal in 17 patients (63%) and retro/intra-abdominal in 10 cases; 14 patients (52%) had received prior chemotherapy. Side effects have been reported elsewhere (ASCO 2002, abstract 1609). One year after the end of accrual, 22 patients were still under protocol therapy. A total of 19 patients (70%) have responded to therapy (1CR, 18PR); 2 of these patients progressed after 261 and 323 days of treatment respectively, but were kept on treatment. Five patients had a stabilization of their disease, ongoing for 280, 371 and 391 days in 3 cases. The 3 other patients had progressive disease at the first disease evaluation (8 weeks). The 1-year estimate (Kaplan-Meier) of progression free survival is 73% (s.e.: 9%). So far, 2 patients have died (both from progression). The 1-year survival estimate is 96% (s.e.: 4%).
*Conclusions:* These results indicate that the activity of imatinib in GIST is long lasting. STBSG has subsequently randomized 946 patients in a phase III trial to compare the activity of the drug administered at 400mg/day and 400mg b.i.d.

**Young Investigator Award Winner**

Molecular Targeting Of Pdgfb By Imatinib Mesylate In Dermatofibrosarcoma Protuberans
[Abstract ID: 25]

**Category:** Medical Oncology
**Presentation:** Young Investigator
**Authors:** Scott M Schuetze[1], Brian P Rubin[1], Janet F Eary[1], Thomas H Norwood[1], Sohail Mirza[1], Ernest U Conrad III[1], James D Bruckner[1]
**Author Institutions:** [1]University of Washington, Seattle, Washington, United States
**Presenter:** Scott M Schuetze
sschuetz@fhcrc.org
**Correspondent:** Scott M Schuetze
sschuetz@fhcrc.org
Seattle Washington United States 98109
Ph: 206 288–2052
Fax: 206 288–2042

*Objectives:* Dermatofibrosarcoma protuberans (DFSP) are highly invasive dermal mesenchymal neoplasms that occasionally metastasize. Standard chemotherapy has not been effective. Activation of the platelet derived growth factor B (PDGFB) receptor, a transmembrane kinase, by aberrant expression of platelet derived growth factor B is central to the pathogenesis of DFSP. We treated a patient with unresectable, metastatic DFSP with imatinib mesylate to investigate the response of this malignancy to inhibition of the PDGFB pathway.
*Methods:* A patient with metastatic DFSP received imatinib mesylate twice daily. Tumor response to therapy was assessed using fluorodeoxyglucose-positron emission tomography, magnetic resonance imaging, immunohistochemistry and standard pathology.
*Results:* The patient tolerated twice daily ingestion of imatinib mesylate. After two weeks of treatment, fluorodeoxyglucose uptake in the DFSP fell to background levels. Tumor volume progressively shrank over four months by more than 75% allowing for complete resection of the residual mass. Histologic evaluation revealed a complete pathologic response to treatment. Adjuvant therapy with imatinib mesylate was not used. The patient remains free of tumor more than nine months after surgery.
*Conclusions:* Imatinib mesylate appears to be highly active in DFSP when administered twice daily. The optimal duration of therapy to obtain a complete response is not known, but in this patient, four months of treatment resulted in a complete histologic response and allowed for complete resection of the residual mass. This result demonstrates that therapies targeting specific oncogene pathways central to the pathogenesis of a neoplasm can result in dramatic therapeutic effects.

Gleevec Therapy In C-kit Negative Soft Tissue Sarcomas: A Molecular Rationale.
[Abstract ID: 57]

**Category:** Medical Oncology
**Presentation:** Oral
**Authors:** Dafydd Gareth Thomas[1], Thomas J. Giordano[1], Robert S. Benjamin[2], Dennis A. Priebat[3], Brian L. Samuels[4], Sarah S. Bacus[5], Laurence H. Baker[1]
**Author Institutions:** [1]University of Michigan Cancer Center, Michigan, United States; [2]UT MD Anderson Cancer Center, Texas, United States; [3]Washington Cancer Institute, Washington, D.C., United States; [4]Lutheran General Cancer Care Center, Illinois, United States; [5]Ventana Medical Systems Inc, Illinois, United States **Presenter:** Dafydd Gareth Thomas
Thomasda@umich.edu
**Correspondent:** Dafydd Gareth Thomas

Thomasda@umich.edu
Ann Arbor Michigan United States 48109–0602
Ph: 734.763.2475
Fax: 734.647.1358

*Objectives:* Imatinib mesylate (Gleevec) therapy has revolutionized the treatment of c-KIT positive soft tissue sarcomas (STS) such as GIST. The North American branch of the Connective Tissue Oncology Society is currently conducting a phase II trial of Gleevec in patients with advanced non-GIST STSs. Recently, a patient with advanced Malignant Fibrous Histiocytoma (MFH) responded dramatically to Gleevec therapy. Immunohistochemical analysis of the resected tumor demonstrated the absence of c-KIT and the presence of PDGFR a and its ligand PDGF-A. The phosphorylated form of AKT was also present. PDGFR a and b are membrane bound receptor tyrosine kinases (RTK), which are thought to be alternate targets for the RTK inhibitor, Gleevec. The genomic sequence for these RTKs share extensive homology with both c-KIT and c-ABL, especially in the region coding for the ligand-binding domain. PDGFR a and b, c-KIT and c-ABL are all strongly inhibited by Gleevec. AKT is a cytoplasmic serine/threonine kinase, which is a common target for RTK phosphorylation. It is involved in the regulation of cell survival.
*Methods:* In order to further determine which patients would benefit from empirical Gleevec therapy, sections of a tissue microarray (TMA) with multiple cores from eight different STS subtypes (rhabdomyosarcoma (n=15), leiomyosarcoma (n=8), liposarcoma (n=10), angiosarcoma (n=8), MFH (n=16), GIST (n=5), synovial sarcoma (n=12), and fibrosarcoma (n=11)) were stained using routine immunohistochemical stains for PDGFR a and b, c-KIT and AKT. Sections were also stained with antibodies specific for the phosphorylated form of AKT.
*Results:* Analysis of the data indicates that although PDGFR a and b are ubiquitous in distribution amongst STS, c-KIT immunoreactivity was only observed in GISTs, synovial sarcomas and angiosarcomas. AKT immunoreactivity was observed in 68 of 85 STS (80%). The phosphorylated form of AKT was seen in 68%, ranging from 36% in fibrosarcomas to 87.5 % in MFH.
*Conclusions:* These results suggest that adjuvant therapy with Gleevec is may be useful in c-KIT negative STSs, where activated forms of AKT is present. The results also provide a molecular rationale for the dramatic response seen in the c-KIT negative MFH patient undergoing therapy with Gleevec.

### Saturday, November 2

### Approach To Prosthetic Reconstruction In Sternal Sarcoma [Abstract ID: 40]

**Category:** Surgery
**Presentation:** Oral
**Authors:** Michele Rocca[1], Antonio Briccoli[1]
**Author Institutions:** [1]Department of General Surgery Rizzoli Orthopaedic Institute Bologna, Italy **Presenter:** Michele Rocca
michele.rocca@ior.it
**Correspondent:** Michele Rocca
michele.rocca@ior.it
Bologna Italy 40136
Ph: 390516366222
Fax: 39051331710

*Objectives:* We performed a retrospective analysis of our experience in chest wall reconstruction after sternectomy for high grade sarcoma. In particular we evaluate a technique of prosthetic reconstruction, where wide resections always including the chest wall are mandatory.
*Methods:* Sternal and chest wall reconstruction were performed in fifteen patients (7 chondrosarcoma, 2 osteosarcoma, 1 angiosarcoma, 1 Ewing's sarcoma, 2 radiation-induced sarcoma, 1 liposarcoma, 1 malignant fibrous histiocytoma). After resection of the primary tumor, a tailored Marlex mesh was anchored to the margins of the wall defect. One or two mouldable metallic plates were fixed either to the remaining rib or to the clavicular stumps and to the residual sternum supporting the mesh, avoiding chest wall volet. Pedicled muscle flaps were always associated to complete reconstruction.
*Results:* Perioperative mortality was 6.6% (1 case). The mean time of postoperative intubation was 8 hours (range 0–18). During postoperative chemotherapy two patients presented a wound infection, healed after local debridement. The margins achieved by "en bloc" resection of the entire tumor (mean resected skin area 99.7cm2) was wide in 12 patients and marginal in 3 (Enneking's surgical staging system). At a mean follow-up of 26.7 months (range 68–12) 13 patients were alive (11 continuously disease free, two alive with distant metastases or local relapse); two patients died. All patients recovered their normal lifestyle.
*Conclusions:* The authors believe that the reconstruction technique adopted is feasible and favorable due to its short hospitalization, good local control, and because it prevents prolonged post-operative mechanical ventilation and future restriction-related working incapacity.

### Autobiological Reconstruction With Preserved Joint Function After Surgical Treatment Of Bone Sarcomas In Children [Abstract ID: 42]

**Category:** Surgery
**Presentation:** Oral
**Authors:** Orjan K Berlin[1], Peter Bergh[1], Bjorn Gunterberg[1], Jonas Lundberg[2], Hans Mark[2]
**Author Institutions:** [1]Department of Orthopaedics Sahlgren University Hospital Goteborg, Sweden; [2]Department of Plastic Surgery Sahlgren University Hospital Goteborg, Sweden
**Presenter:** Orjan K Berlin
berbiz@algonet.se
**Correspondent:** Orjan K Berlin
berbiz@algonet.se
Goteborg Sweden S-413 45
Ph: +46–31–828284
Fax: +46–31–410402

*Objectives:* After limb-salvage surgery in young children with bone sarcomas our preferences are biologic reconstructions using autologous vascularized fibula grafts. This report describes 3 children in whom the joint function could be preserved in the knee, hip and shoulder joints respectively.
*Methods:* All 3 patients had clinically good response to preoperative chemotherapy. A 9-year-old girl with an osteosarcoma of the distal femur was reconstructed with dual parallel fibula grafts with the proximal fibula epiphysis towards the knee. The lateral collateral ligaments (LCL) were sutured to the cruciate ligaments. Soft tissue resection was kept to a minimum since it was a stage IIA lesion.
A 9-year-old girl with a stage IIB Ewing sarcoma of the right proximal femur was reconstructed with 2 fibula grafts; one proximal fibula replaced the femoral head and was inserted into the remaining femur, the other graft served as support. The hip-joint capsule was sutured like a pouch around the fibula head.
A 6-year-old girl with a stage IIB telangiectatic osteosarcoma of the left proximal humerus was reconstructed with a single fibula graft. The origin of the long biceps tendon was sutured to the LCL, and the rotator cuff was sutured as a pouch around the fibula head.
*Results:* 19 months postoperatively the knee reconstruction is ambulatory with a (MSTS) score of 10/30. At 33 months the hip reconstruction is ambulatory with a score of 13/30. At 29 months the shoulder reconstruction has an excellent function with a score of 27/30.
*Conclusions:* Until now the follow-up results appear encouraging.

Endoprosthetic Replacement Of Distal Humerus After Tumor Resection
[Abstract ID: 74]

**Category:** Surgery
**Presentation:** Oral
**Authors:** Ashwin Kulkarni[1], Fabrice Fiorenza[1], Rob J Grimer[1], Abesegun Abudu[1], Simon R Carter[1], Roger M Tillman[1]
**Author Institutions:** [1]The Royal Orthopaedic Hospital Birmingham, United Kingdom
**Presenter:** Fabrice Fiorenza
Fabrice.Fiorenza@chu-limoges.fr
**Correspondent:** Rob J Grimer
rob.grimer@btopenworld.com
Birmingham United Kingdom B31 2AP
Ph: 44 1216854000
Fax: 44 1216854146

*Objectives:* The purpose of this study was to determine the outcome of patients after distal humerus reconstruction for bone tumors using endoprosthetic replacement (EPR).
*Methods:* 10 patients were retrospectively reviewed after resection of a primary or metastatic tumor of the distal humerus between 1970 and 2001.They all had a custom made distal humerus EPR. No patients was lost to follow up. The Toronto Extremity Salvage score (TESS) was used to assess function in patients still alive.
*Results:* There were 4 male and 6 female patients, with ages ranging from 15 to 76 years. The period of follow up ranged from 5 months to 31 years. 8 patients had primary tumors and 2 had secondary tumors. 4 out of 10 patients developed metastasis and died 12 to 71 months after the operation. None of the 10 patients had local recurrence, infection, amputation or permanent nerve palsy. Average flexion deformity was 15 degrees (0–35) and average flexion of these patients was 115 degrees (110 – 135). There were 3 revisions at 48, 56 and 366 months for aseptic loosening. There were 3 rebushings of the plastic inserts at 62,78 and 113 months. Two of the three rebushings were done after revision of the humeral component at 6 months and 30 months. The average TESS score for these patients was 72.91 (29.2 to 93.33).
*Conclusions:* Custom made EPR for distal humeral tumors are an effective way of replacing the diseased bone leading to a reasonable level of function and an acceptable failure rate.

Survivorship Analysis Of 141 Modular Metallic Endoprostheses
[Abstract ID: 26]

**Category:** Surgery
**Presentation:** Oral
**Authors:** Erik N. Zeegen[1], Luis Aponte[1], Francis J. Hornicek[1], Mark C. Gebhardt[1], Henry J. Mankin[1]
**Author Institutions:** [1]Massachusetts General Hospital, MA, United States
**Presenter:** Mark C. Gebhardt
mgebhardt@partners.org
**Correspondent:** Erik N. Zeegen
ezeegen_00@yahoo.com
Boston MA United States 02114
Ph: (617) 724–3700
Fax: (617) 726–6823

*Objectives:* To evaluate the survival rates and clinical results of modular endoprostheses in patients who have had resections for musculoskeletal tumors and some non-tumorous conditions.
*Methods:* We retrospectively reviewed the records of 141 patients in whom a modular endoprosthesis was implanted over the past 8 years. Statistical data was compiled using Kaplan-Meier survival analysis and a multivariate regression analysis was performed to identify independent risk factors. Failure was defined as need for revision of the majority of the prosthetic components or complete removal of the implant. Clinical results were graded according to a scoring system previously reported.
*Results:* There were 67 males and 74 females. The average age at the time of surgery was 48.9 years. There were 13 failures yielding an overall implant survival of 91%. Based on Kaplan-Meier estimates, the endoprosthetic survival rate was 88% at 3 years and 76% at 5 years; per location, it was 100% for proximal humerus, 100% for proximal femur, 87% for modular knees, and 53% for total femoral implants at 3 years ($p = 0.03$). The failure rate of failed allografts converted to an endoprosthesis was 18% versus 6% for primary endoprostheses ($p = 0.08$). However, multivariate analysis showed that only location, infection, and loosening were independent risk factors for prosthesis failure. Clinical scores were 74% good-excellent and 26% fair-poor. Ten patients died (9 of metastatic disease, 1 from peri-operative complications).
*Conclusions:* We feel that our experience, albeit short in follow-up, is similar to other endoprosthesis survivorship reports in the literature at early time points.

Low Complication Rate With Limb-sparing Resection And Endoprosthetic Reconstruction: Survival Analysis Of 251 Patients And Analysis Of 20-year Experience
[Abstract ID: 59]

**Category:** Surgery
**Presentation:** Oral
**Authors:** Felasfa M Wodajo[1], Kristen Kellar-Graney[1], James C Wittig[1], Robert M Henshaw[1], Martin M Malawer[1]
**Author Institutions:** [1]Washington Cancer Institute, DC, United States
**Presenter:** Felasfa M Wodajo
felasfa@earthlinl.net
**Correspondent:** Felasfa M Wodajo
felasfa@earthlinl.net
Washington DC United States 20010
Ph: 202–877–3970 Fax: 202–877–8959

*Objectives:* In order to evaluate patterns of complications and prosthetic survival after endoprosthetic reconstruction for bone tumors, the authors reviewed all procedures performed over a 20-year period at a single institution.
*Methods:* Methods
Patients who underwent one or more procedures with a segmental endoprosthesis, except for saddle prostheses, are reported. Prosthetic failure is defined as removal of a cemented component. Kaplan-Meier survival analysis, interval to complication and the number of operations per patient are calculated.
*Results:* Results
A total of 251 patients underwent surgery. Of these, 197 patients (78%) underwent only one surgery. The remaining 54 underwent a mean of 3.5 procedures.

Complication
Type n Follow-up
(months) Interval between first and second surgery # Surgeries
Prosthesis failure
mechanical 20 121.2 45.5 3.6 11 (7 custom)
soft tissue
(superficial) 10 35.1 44.1 3.0 0
soft tissue
(deep) 5 76.6 44.1 3.0 0
infection 11 79.3 14.2 4.6 6
dislocation 2 29.8 7.8 3.0 0
other 6 81.3 7.8 3.0 1
Overall 54 85.4 25.5 3.5 18
% (÷251) 21.5% 7.2%

**Anatomic location vs. Prosthetic Types**:

| | D. Femur | P. Tibia | P. Humerus | P. Femur | Scapula | T. Femur | Other | Total |
|---|---|---|---|---|---|---|---|---|
| Custom | 19 | 9 | 6 | 9 | 11 | 9 | 4 | 78 |
| Modular | 74 | 31 | 32 | 24 | 0 | 7 | 5 | 173 |
| Expandable | 0 | 0 | 0 | 0 | 0 | 0 | 0 | 11 |
| Total | 93 | 40 | 38 | 33 | 11 | 16 | 9 | 251 |

*Conclusions:* Overall prosthetic survival (92.8%) has proven to be greater than most surgeons anticipated in the early days of limb-salvage surgery. At an average follow-up of 7 years, 67% (36/54) patients who required reoperation were able to retain their prostheses. All patients with soft tissue complications were salvaged with an average of 3 procedures, while 45% (5/11) of patients with deep infections were salvaged. Prosthetic failure due to mechanical complications diminished significantly from 64% (7/11) to 36% (4/11) with introduction in 1988 of modular prostheses. Meticulous attention to technique and aggressive management of soft-tissue complications leads to excellent prosthetic survival.

Is There A Case For A Randomized Clinical Trial For Treatment Of Giant Cell Tumours?
[Abstract ID: 77]

**Category:** Surgery
**Presentation:** Oral

**Authors:** Robert Grimer[1]
**Author Institutions:** [1]Royal Orthopaedic Hospital, United Kingdom
**Presenter:** Robert Grimer
rob.grimer@btopenworld.com
**Correspondent:** Robert Grimer
rob.grimer@btopenworld.com
Birmingham United Kingdom B31 2AP
Ph: (+44) 121 685 4150
Fax: (+44) 121 685 4146

*Objectives:* The role of adjuvants in preventing local recurrence following curettage of giant cell tumour of bone (GCT) remains controversial. We have reviewed our experience of curettage alone to fuel the debate.
*Methods:* All patients treated for primary giant cell tumours of bone over a 27 year period by detailed curettage with a high speed burr were reviewed. Risk factors for local recurrence were identified.
*Results:* Of 137 with previously untreated GCT of bone, 29 had a pathological fracture. Campanacci grading showed that 14 were grade I, 47 were grade II and 52 were grade III Following curettage, 26 patients (19%) developed local recurrence (LR) at a mean of 22 months. Size, site and fracture were not related to LR. For intraosseous tumours (grades I and II) the LR rate was 7% whilst for extraosseous tumours the LR rate was 29%. Of the 26 LRs, 16 had a further curettage of whom 10 were cured by one curettage and 4 following a third curettage. The success rate of curettage was 81% with one operation, 88% with two and 91% with three curettages. The mean MSTS functional score in these patients was 94%.
*Conclusions:* The literature suggests an overall LR of 27% for curettage and 19% when an adjuvant is used. With cryotherapy an LR of 4% was achieved. Local control of GCT of bone is not yet resolved. The role of adjuvants has not been proved by the published literature and an international prospective randomized trial is recommended.

